# Graduate perspectives on a transnational medical programme: a qualitative study of career impact

**DOI:** 10.1186/s12909-026-09693-8

**Published:** 2026-06-30

**Authors:** Gamarelanbia Yousif, Ashraf Shoma, Emad Samaan, Hend Mahmoud Hassan AboElatta, Lucie Byrne-Davis, Jo Hart

**Affiliations:** 1 Division of Medical Education, University of Manchester, Manchester, UK; 2 University of Mansoura, Mansoura, Egypt

**Keywords:** Graduate perceptions, Transnational medical education, Clinical preparedness, International education partnerships, Health professions education.

## Abstract

**Background:**

Transnational education (TNE) represents an evolutionary development in internationalisation, involving the movement of institutions or their programmes rather than student mobility. Influenced by the ambitions and strategies of sending and receiving countries, TNE offers distinct values and benefits to all stakeholders. Most previous research has focused on the successes and challenges of TNE programmes from the perspectives of programme directors, administrators, and educators, but less is known about graduates’ experiences. This study explored graduates’ perceptions of a UK-affiliated transnational medical programme in Egypt and its impact on their personal and professional development and careers.

**Methods:**

We conducted a qualitative study with graduates from a long-established transnational medical programme delivered under a UK university’s licence in Egypt. We purposively sampled graduates to maximise variation in age, gender, nationality, professional status, current job, and country of work. We conducted semi-structured online interviews via Zoom, audio-recorded them, and transcribed the recordings. We adopted a constructivist/interpretivist approach and conducted reflexive thematic analysis, ensuring reflexivity was maintained throughout the study.

**Results:**

Twenty-three graduates were interviewed for an average of 45 min; 14 were female and 9 were male, with ages ranging from 23 to 33 years. We identified six overarching and interconnected themes: (1) Personal and professional transformation through generic skills and critical thinking; (2) An integrated and well-organised curriculum delivered through case-based discussions; (3) Clinical preparedness fostered by small class sizes and early clinical exposure; (4) International career readiness supported by English language proficiency, exchange programmes, relevant examination formats, and multinationalism; (5) Supportive teaching and leadership staff from both institutions; (6) Immersive research experience.

**Conclusions:**

We explored insightful perspectives and experiences of the graduates. We found that an integrated, internationally focused curriculum, delivered and overseen by effective and supportive staff, prepares graduates to serve locally and internationally with notable resilience and confidence. These firsthand insights contribute to the limited literature on graduates’ perspectives in TNE, particularly in health professions education (HPE), and offer practical, achievable recommendations for curriculum, assessment, student support, and the learning environment. Further research exploring staff and institutional viewpoints is necessary to gain a more comprehensive understanding.

**Supplementary Information:**

The online version contains supplementary material available at https://doi.org/10.1186/s12909-026-09693-8.

## Background

The internationalisation of health professions education (HPE) is a broad, inclusive concept, interpreted and implemented differently depending on stakeholder motives and contextual factors [1–3]. One major dimension of internationalisation is academic mobility, which includes the movement of students and staff across countries for educational purposes. Students’ mobility to other countries to pursue high-quality education and degrees offers authentic and immersive experiences [4]. Still, it is expensive, faces numerous logistical and political barriers, and is accessible only to a minority of students [5, 6]. Staff mobility has been implemented in various arrangements, including fly-in faculty [7] and, more recently, online ‘mobility’. Staff mobility can support teaching, research, designing, and evaluating various curricula, but it faces numerous national and international challenges [8]. International academic mobility is an integrated term for the movement of both students and staff [9]. In contrast, the mobility of institutions or their programmes is referred to as transnational education (TNE) [7, 10, 11].

Transnational education is a rapidly growing form of internationalisation because it is flexible and offers a range of programmes to suit different stakeholders [7, 12]. It has various models, such as franchising and licensing programmes, partnership programmes, international branch campuses (IBC), joint colleges/universities and different distance education models [7]. Curriculum-based programmes, such as franchising and licensing programmes, are more common in the UK, the Netherlands and Australian institutions, whereas USA institutions focus more on IBCs [13, 14]. Distance education may be considered a distinct type of TNE or simply a pedagogical method [7]. International partnerships in education as part of TNE include joint degree programmes, dual/double degree programmes, multiple degree programmes, twinning/articulation programmes and top-up programmes [7]. International medical programmes typically use English as the medium of instruction, deliver internationally oriented integrated curricula and enrol international students [15, 16]. Some institutions offering these international programmes also run conventional versions for domestic students in their native language [17].

In HPE, TNE is increasingly recognised as a complex, evolving, and diverse field spanning professional and regulatory boundaries, often intersecting with educational, health systems, licensure, and cultural contexts [7, 18]. These complexities are especially pronounced in clinical education, where language barriers, local healthcare practices and differing competency requirements present unique challenges. Unlike traditional education models, TNE in HPE requires alignment with the expectations of multiple stakeholders, including accrediting bodies, institutional partners, and students. The need for graduates to be both clinically competent and culturally responsive across global and local settings adds further complexity [18, 19]. In Egypt, the growth of transnational medical education is driven by internationalisation efforts, including university collaborations, global curricula, quality assurance, and student and staff mobility. While these initiatives aim to prepare students for global healthcare, they can create tension between global and local priorities. It is of utmost importance to explore graduates’ experiences within this context.

Previous studies have primarily focused on the perspectives of programme directors, administrators, and educators [18, 20, 21], especially from the home institutions (institutions that design these curricula, which are primarily located in the West). In some studies, host teachers are satisfied with these international curricula, but they express concerns about their relevance to local contexts, which require both content and pedagogical adaptations [22]. Quantitative measures such as satisfaction and students’ enrolment percentages have been studied without considering their effects on personal and professional development, international career readiness and cultural adaptation [10]. Some studies focus on specific aspects of these programmes, such as the importance of formal career guidance and the appropriate level of clinical preparedness [17]. Some studies address challenges related to accreditation, navigating various health systems, and job opportunities [17]. While internationalisation and transnational medical education aim to expand access to global educational opportunities, concerns remain regarding cultural relevance, adaptation to diverse educational and healthcare systems, and the long-term impact of these programmes on graduates’ personal and professional development. However, little is known about how these programmes shape graduates’ long-term career readiness, professional identities, and experiences across diverse healthcare and cultural contexts.

In this study, we aimed to explore graduates’ perceptions of a transnational medical education programme delivered through an international partnership using a licensed curriculum. Adopting a constructivist/interpretivist approach [23, 24], we sought to understand their diverse lived experiences and the perceived impact of the programme on their personal and professional development. Guided by transformative learning theory [25], which explains how challenging educational and cultural experiences can reshape perspectives, identities, and professional development, we explored how graduates understood the programme’s influence on their personal transformation, career readiness, and professional trajectories. The theory informed our conceptualisation of learning as a potentially transformative process and guided our interpretation of participants’ experiences across academic, cultural, and clinical contexts.

## Methods

### Study context

A collaborative licensing programme was established in 2006 between a UK university and an Egyptian university [26]. The programme follows a five-year integrated spiral curriculum structured around problem-based learning (currently team-based learning), incorporating biopsychosocial aspects of health and focusing on competencies outlined by the UK General Medical Council [27]. The curriculum was designed by the UK university and licensed to the Egyptian university, while the UK partner provides oversight of programme delivery and quality assurance. This model differs from the more traditional, lecture-based, discipline-segregated approach used in some Egyptian universities.

Internationalisation within the programme is reflected through the implementation of an internationally oriented curriculum, bilateral faculty visits, external supervision, and student exchange opportunities. The programme has grown from 60 to approximately 400 students per cohort, with around 50% of students originating from 45 countries [26]. To date, the programme has produced 15 graduating cohorts [26]. The programme follows a competency-based curriculum model aligned with international medical education frameworks [28, 29]. As with other transnational medical education programmes, it operates by balancing international standards with responsiveness to local healthcare needs and workforce priorities.

### Study design

We conducted an exploratory, qualitative study within a constructivist/interpretivist paradigm to interpret and construct meaning from participants’ firsthand insights and lived experiences during and after the programme [23, 24]. This paradigm aligns well with the study’s aims and objectives to explore diverse graduate perspectives, each reflecting subjective, contextual realities. We selected semi-structured interviews for data collection to facilitate open yet focused conversations, allowing greater flexibility and exploration while remaining within the study’s scope. We chose reflexive thematic analysis because it aligns closely with our chosen paradigm and the exploratory nature of our inquiry, fostering a more inductive approach to analysis [30]. Furthermore, it emphasises participants’ lived experiences and critical reflection, enabling the researcher to derive and develop meaningful themes through an active participant-centred approach. Transformative learning theory provided an additional conceptual lens for understanding how graduates interpreted and reflected on their educational experiences, such as transformative changes in perspectives, professional identity, and personal and professional development [25].

### Recruitment strategy

The Egyptian university emailed its alumni and advertised on its Facebook page. Both the advertisement and the email contained a link to a set of brief questions. Although the study invitation and Qualtrics link were disseminated via these channels, the research team was unable to ascertain the number of graduates who received or viewed the invitation; therefore, the overall number of eligible graduate contacts and the response rate could not be determined. Participants were asked to click on a link to enter their email addresses. The researcher sent the study details, including the participant information sheet, interview guide questions (Supplement 1), and consent form, to anyone who submitted their email address. We made three email reminders every two weeks to encourage participation. The researcher and participants signed the written consent form before the interview.

### Participants and sampling

Eligible participants included graduates from different programme cohorts who were currently working or undertaking further professional training in diverse settings.

We used purposive sampling to select graduates from different age groups, genders, years since graduation, nationalities, professional statuses, specialities, and current countries of employment. This strategy aimed to ensure a broad and balanced range of perspectives that reflect the programme’s role and impact across various graduate cohorts. Our sampling approach was guided by information power and data sufficiency, ensuring adequate depth, richness, and variation in participants’ reflections. Recruitment and data collection continued until sufficient diversity, depth, and recurrence of themes were achieved, with subsequent interviews contributing limited new insights [30].

### Data collection

The primary researcher (GY), a medically qualified male PhD student with experience in education, conducted all semi-structured interviews via Zoom. Each interview lasted 45–60 min and was audio-recorded. Participants were not previously known to the researcher. A pilot interview was conducted with a graduate from the programme (not included in the main study), which helped to refine the interview guide questions and improve their clarity and flow. During interviews, participants responded to open-ended questions (probing questions) about the programme and its career impact. Recordings were transcribed using Otter.ai and checked for accuracy against recordings by (GY). Identifiable information was removed, and audio files were deleted two weeks after analysis. Field notes were recorded during and after interviews to capture observations, insights, and ideas. Data collection began on May 24, 2024, and ended on December 16, 2024, after reaching data adequacy. We followed the COREQ checklist (supplement 2) to ensure transparency and rigour [31]. Transcripts were not returned to participants for comment or correction. However, during interviews, the researcher summarised key points and reflected participants’ statements to them to ensure accurate understanding and clarification. Participants were encouraged to elaborate, correct, or clarify their responses and were free to decline answering any question. This iterative approach enhanced the credibility and trustworthiness of the data.

### Data analysis

We used Braun and Clarke’s six phases of reflexive thematic analysis because of its flexibility, participant-centred focus, and ability to capture lived experiences [30]. The primary researcher (GY), who was not affiliated with the programme, independently coded all transcripts after repeatedly reading and familiarising themselves with the content. Initial codes were generated inductively from participants’ accounts. Although coding was conducted inductively, theme development and interpretation were informed by concepts from transformative learning theory, including perspective transformation, critical reflection, and personal and professional growth [25]. Subsequently, we organised related codes into potential themes and continuously revisited and compared them internally with their codes and externally across all other themes in the dataset to ensure consistency. Finally, after holding regular meetings, we identified the final themes, supported by representative extracts, which clearly articulated the study’s aims and objectives. These themes were discussed with the supervisory team (JH and LBD), who, although they did not access the raw data, provided methodological guidance and critical feedback. Themes were further refined through regular team discussions involving all co-authors. Collaborators from the programme in Egypt (AS, ES, HA) contributed contextual insights, enriching cultural relevance. This collaborative, reflexive approach enhanced the rigour and credibility of the analysis while reducing bias and positional influence.

### Reflexivity

The primary researcher (GY) contributed an external perspective, as they had no affiliation with the programme. This detachment offered an outsider’s critical viewpoint and facilitated balanced discussions throughout recruitment, data collection, and analysis. The other members of the research team (AS, ES, HA, LBD, JH) were engaged in the programme through teaching or supervisory roles, thereby providing internal insights that enhanced the contextual understanding and interpretation of the findings. The diverse insider-outsider perspectives within the research team particularly influenced the interpretation of issues related to educational culture, professional identity formation, and perceptions of internationalisation.

To address potential positionality, the team maintained a reflexive stance, recognising that different backgrounds, roles, and assumptions might shape interpretation of the study and its findings [30]. Although GY conducted the initial coding and analysis, coding and theme development were regularly discussed with the entire team to challenge assumptions, refine interpretations, and consider alternative explanations throughout analysis. Rather than attempting to eliminate subjectivity, the study acknowledged its role in qualitative interpretation and used reflexive discussions to enhance transparency and credibility [30].

### Ethical considerations

The Research Ethics Committee at the University of Manchester approved the study on 25 March 2024 (Ref: 2024-18888-33997) in accordance with the Declaration of Helsinki. We also obtained a letter of permission from the collaborating institution. Informed consent was obtained from all participants.

## Results

### Demographics of the participants

Of 55 graduates who initially expressed interest in participating in the study, 23 participated in interviews, 4 apologised due to personal and work commitments, and the remainder did not respond to reminder emails. This sample achieved data adequacy and information power, including graduates from different programme cohorts and graduation years, ensuring diversity in age, gender, nationality, and professional roles that broadly reflects the programme’s composition (Table 1).

**Table 1:** Demographic characteristics of study participants

Demographic variable	*N* (%)
Age	23–33 years, (Mean = 27.7)
Gender	Females: 14/23 (60%)
	Males: 9/23 (40%)
Nationality	82% Egyptian, 9% Bahraini, 9% Malaysian
Graduation year	2013–2023 (95% graduated between 2018–2023)
Professional status	40% registrars, 30% senior house officers (SHO), 10% researchers, 10% interns and 10% lecturers
Professional domain	80% in clinical specialities and 20% in education and research
Current setting	90% in hospitals and primary care, 10% in higher education
Country of work	43% in Egypt, 35% in the UK, 10% in the USA, 4% in Saudi Arabia, 4% in Bahrain, and 4% in Malaysia

Data are presented as n (%) unless otherwise indicated

### Themes and subthemes

We identified six interconnected themes reflecting different but related dimensions of graduates’ experiences and perceived programme impact, including personal and professional transformation; well-organised and integrated curriculum; clinical preparedness; international career readiness; effective and supportive teaching and leadership staff; and authentic research experience. Although presented separately for clarity, these themes were highly interconnected and collectively shaped graduates’ personal and professional transformation and career readiness. Together, these themes illustrate how the programme influenced graduates’ academic, professional, intercultural, and career development across diverse contexts.

### Theme 1: Personal and professional transformation

This theme reflects broader changes in graduates’ personal growth, self-confidence, professional identity, generic skills, and ways of thinking and learning resulting from exposure to diverse educational and cultural experiences. Most participants described becoming more personally and professionally resilient through developing generic skills such as critical thinking and problem-solving. The shift from teacher-centred to student-centred learning was transformative, particularly for students from diverse educational backgrounds. Group work and communities of practice—through PBL, clinical training, and research activities like poster design—enhanced this development. The programme fostered inclusive academic and social spaces, enabling collaboration across cultures. Most graduates felt well prepared, both clinically and culturally, to serve in local and global contexts.

### Enhancement of graduates` competence and confidence

Through PBL and other teaching methods in clinical training, students developed different study skills in communication, presentation and public speaking.

‘I was shy, so more communication between […] colleagues, […] doctors and patients helped me […] Designing posters and […] discussing the objectives […] helped me gain confidence […] I see communication […] as the key.’ P18.

This illustrates how interactive and discussion-based learning methods contributed to graduates’ confidence, clinical communication skills and professional development.

One participant requested more sessions on communication and interviewing skills.

‘I think the programme can improve communication skills. […] When I started in the UK, I could manage patients well but had difficulty presenting cases compared to UK doctors. […] ‘Sessions should teach students proper interviews, calmness, and presentation skills.’P2.

### Cultivation of critical thinking

By combining their clinical reasoning with reflection, most participants felt well prepared to manage clinical complexity and uncertainty.

‘What I noticed lately is that my thinking is different. The way I analyse medical information is different. I learned not just how to memorise but also to think about […] medical knowledge and analyse it.’ P12.

This reflects how participants perceived the programme as a transformational factor, moving from didactic teaching and learning towards more critical and reasoning-based approaches to medical education.

### Theme 2: Integrated and well-organised teaching approach

#### Case-based discussions

Through a single case, students acquire integrated knowledge from the basic, behavioural, social, and clinical sciences, and they experience greater integration as they progress through the spiral curriculum. Students address learning outcomes of specific themes each semester through both self-directed study and group discussions. There is always scope for students to go beyond these primary outcomes to foster greater innovation and flexibility.

‘Case-based discussions felt like a puzzle that came together. I don’t like being passive or treated as a vessel for information. […] Case-based discussions treated us as people with functioning brains.’P17.

However, some participants requested that the programme allocate more time to the basic sciences.

‘Students from conventional programmes have detailed knowledge of all basic sciences. I would like the programme to give more focus on basic sciences such as pathology.’P16.

A few participants suggested training for PBL tutors to enhance their facilitative skills.

‘I think a few doctors need to be more trained on the style of the PBL. Some doctors activate brainstorming, and the discussion is interesting. Other PBL sessions are boring. It is not always the doctors’ fault; sometimes students are not engaged.’ P14.

#### Good academic and social balance

The programme is well-distributed and structured to provide a balanced learning experience.

‘We have more social life because we have more time for extracurricular activities […] and longer vacations, so we are not burned out’. P9.

### Theme 3: Clinical preparedness

This theme focuses on graduates’ perceptions of how they integrate theoretical knowledge, clinical competence, and communication skills, and their preparedness to practise patient-centred care within a biopsychosocial model of consultation. Clinical preparedness, a key graduate competency, was strongly supported by small class sizes and early, structured clinical exposure. Most participants reported that these features helped them apply theoretical knowledge from lectures and PBL in real settings, reinforcing professional skills and fostering patient-centred care. Most participants felt confident transitioning into clinical roles locally and globally, as early immersion reduced fear and built familiarity with clinical environments.

#### Small class size

Small class sizes maximise learning potential by creating a conducive learning environment for theoretical and practical training.

‘The small class size helped us interact as a team, get to know each other and develop our collective thinking. So, it was perfect. P11.

#### Early clinical exposure and patient-centred approach

Early clinical experience in hospitals or community settings helps students apply their knowledge, boosting their confidence and competence.

‘I’m more confident and calmer in dealing with patients because of early clinical experience. I don’t feel fear or intimidation in clinics or wards as I’ve been dealing with patients for at least four years.’P20.

However, one participant requested more procedural training, stating:

‘The programme needs to do more with the procedures daily, such as taking blood samples, venepuncture, arterial blood gas, inserting a cannula and nasogastric tube.’ P23.

To improve the clinical training, one participant stated, ‘Assigning clear roles to students in the hospital would help. […] I prefer a maximum of three students per resident. […] Sometimes we go to the hospitals just to take a history and leave.’ P13.

This reflects the importance of structured clinical roles and supervised participation in enhancing clinical learning experiences.

### Theme 4: International career preparation

This theme specifically relates to graduates’ readiness to work competently and resiliently in international professional contexts characterised by diverse cultural, educational, and healthcare practices. Most participants viewed the programme as a key foundation for international career readiness, supported by four main factors. Exchange programme opportunities aided early-career navigation, while assessments such as multiple-choice questions (MCQs) and objective structured clinical examinations (OSCEs) aligned with international licensing standards. A diverse student body from over 45 countries fostered cultural competence and a global outlook. English as the sole language of instruction enhanced communication skills and preparedness for global education and healthcare systems.

#### Exchange and placement programmes

Through exchange programmes, participants navigated different cultures, educational and health systems and provided valuable feedback to their programme upon their return.

‘The exchange programme challenged my perspective on medicine, and the teaching style impacted me greatly. […] Based on my feedback, a lot changed in the programme.’P19.

This demonstrates how international exposure fostered both intercultural learning and graduates’ commitment to and contributions to programme improvement.

#### Relevance of assessment approaches to international licensing exams

The assessment strategy prepares graduates for international licensing and professional exams.

‘The programme helped me pass the PLAB [Professional Linguistic Assessment Board] exams at the first trial, and I also cleared MRCS [Membership of the Royal College of Surgeons]’. P15.

Although most participants were very pleased with the outcomes of the programme’s assessment, a few participants requested revision of the construction of the MCQs and the delivery and scoring of OSCE exams, as illustrated in the following quotes:

‘The curriculum is perfect, and the approach and delivery are perfect. But we don’t have an MCQ bank for practice. The MCQ exams were tough because they sometimes asked unreasonable questions. […] construction of some questions must be reviewed.’ P17.

‘Trying some way to standardise OSCEs to avoid having the inter-relational impact on the exam. I cannot think of a way right now. I don’t know if a chaperone or external examiners would work, but I think it would work.’ P6.

#### Multi-nationality and Inclusivity of the Programme

The presence of multiple nationalities in the programme cultivates cultural sensitivity and prepares students for diverse work environments.

‘Nobody felt like an outsider in the programme, which is good. And being taught in English and handling everything in English was good because it didn’t make you feel different when you started work in the UK, for example.’ P16.

However, some participants, namely international graduates, requested linguistic support during their clinical placements.

‘The programme needs to improve in supporting teaching Arabic for us, which I didn’t get.’ P5.

#### Improvement of the English language

Participants acknowledged that the programme operates solely in English across clinical and academic settings, facilitating communication and connection among people from all nationalities and cultures.

‘Learning in English stands out for me because it is the international language of medicine. […] It helps communication, research, and participation in multinational programmes.’ P19.

Participants regarded English-based instruction as a foundational framework for facilitating international communication, dissemination, and career mobility.

### Theme 5: Accessible and supportive leadership and teaching staff

This theme was raised by all participants, who praised the academic and social support received from staff. Participants described leaders and teachers as approachable, responsive, and committed to mentoring and career development. They appreciated their openness to feedback and efforts to create inclusive and supportive learning environments.

‘I value my teachers in the programme very much. They don’t just want to give you hard exams but genuinely want to teach and support you, and this is something I appreciate.’ P20.

Some participants requested the reactivation of clinical placements in the UK, which were suspended during the COVID-19 pandemic.

‘Since our batch arrived in the UK in 2019 during COVID, there have been no clinical placements since then. Administration should restart these programmes, as they provided a valuable experience.’ P10.

One participant recommended clearly communicating the type of programme, degree, and its recognition to prospective students.

‘Some students thought and perceived that the programme is an actual Manchester campus in Egypt.’ P8.

### Theme 6: Authentic research experience

Through integrated research activities, students developed key skills in identifying research questions, reviewing literature, and presenting findings. These skills supported self-directed learning in case-based discussions. Graduates described applying research through posters, essays, and viva voce preparation, with some undertaking authentic projects that led to publications.

‘I guess the Viva part in the final year and posters in the early years give you much confidence going into that after graduation.’ P23.

‘We had workshops on how to conduct and write research, including systematic reviews and research participation.’ P14.

Participants linked these experiences to improved research literacy and self-directed learning skills.

## Discussion

This study deepens the understanding of transnational education by shifting the focus from institutional leaders to graduates. By exploring graduates’ lived experiences, the study highlights how integrated, competency-based curricula were perceived to support preparedness for local and international practice. These approaches align with global competencies endorsed by the World Federation for Medical Education (WFME) and the World Health Organisation (WHO) [28]. This standardisation of global clinical and cultural competencies aims to support the achievement of safe, equitable and universal health coverage [32]. Moreover, these insights from graduates can inform policymakers about potential changes that could improve outcomes of transnational programmes across different levels. In a broader context, the study contributes to the ongoing debate about the opportunities and challenges that imported curricula pose for different stakeholders [10, 15, 18, 20]. Influenced by transformative learning theory [33], which proposes that exposure to challenging experiences/dilemmas can transform individuals’ assumptions, perspectives, and professional identities, and aligned with previous studies [3, 34], graduates articulated how engagement with student-centred learning, problem-based learning, intercultural experiences, and diverse educational settings challenged their pre-existing perspectives and facilitated both personal and professional growth [35]. These experiences appeared to support the development of reflective thinking, confidence, adaptability, resilience, and the formation of professional identity.

Additionally, the programme’s English-language instruction, international exchange opportunities, and multicultural learning environment contributed to graduates’ intercultural awareness and global outlook. These findings also align with principles of andragogy, particularly the emphasis on self-directed learning, active participation, experiential clinical learning, and the practical application of knowledge, which shaped graduates’ learning experiences and preparedness for practice [36, 37].

Case-based discussions that integrate knowledge from basic, clinical, behavioural, and social sciences help students to gradually develop clinical reasoning skills and become reflective learners and practitioners [38–40]. The structured organisation and balanced distribution of curricular elements were perceived to reduce stress and mitigate the risk of burnout [41]. However, some participants expressed a need for greater emphasis on basic sciences to strengthen their foundational understanding. Curriculum developers may need to address this feedback, balancing learners’ needs with the principles of competency-based education, which prioritise integration and applied competence over content coverage.

Two key factors were identified as instrumental in promoting clinical preparedness: small class sizes and early clinical exposure. The former facilitates more intensive theoretical and practical learning environments [42], while the latter fosters confidence and familiarity with healthcare settings by maximising students’ interaction with patients and clinical staff from early in the programme [43]. These features may be particularly valuable for institutions aiming to prepare students for both local and international clinical and non-clinical practices.

However, language and cultural barriers emerged as challenges for non-Arabic-speaking students during clinical placements. While many adopted adaptive strategies—such as relying on peer support or translation tools—these coping mechanisms point to a critical institutional responsibility.

Programmes with a linguistically diverse student body should consider implementing formal support systems, including Arabic language instruction or culturally sensitive communication workshops, to ensure equitable clinical preparation. One study even recommends involving English-speaking patients to facilitate international students’ clinical training [44]. This theme alerts programmes to the cultural and linguistic challenges that international students face in the local clinical environment and outlines the best ways to support them.

Most participants are internationally oriented, especially domestic ones, who join the programme because it prepares them for various international careers. Exchange programmes allow students to explore different cultures, education systems, and healthcare systems, consider potential future careers, and compare their learning experiences with those of their home institution [45]. However, these visits must be inclusive and have clear plans and objectives for both institutions and students [3]. Moreover, the programme operates solely in English, which helped graduates pass language tests such as the Occupational English Test (OET) and the International English Language Testing System (IELTS) with ease. Additionally, they pass international licensing exams, such as the UK PLAB (now the UK MLE), on the first attempt because the assessment formats are similar. The presence of 45 nationalities in one space fostered cultural understanding and tolerance, preparing graduates to live and work with diverse backgrounds.

Aligned with other studies [17, 46], most graduates worked outside the country where they were trained (Egypt), including in the UK, USA, and other destinations. Participants frequently described international career aspirations and perceived the programme as supporting mobility and preparedness for global practice. Some domestic graduates also reflected that graduates within certain cohorts increasingly sought employment abroad after graduation. While these findings may reflect the programme’s perceived contribution to global career readiness, they also raise broader concerns, as discussed in previous literature, regarding health workforce migration and potential brain drain from low- and middle-income countries [18].

Consistent with other studies, the teaching and leadership staff support students at both academic and social levels, facilitating their development through curricular and extracurricular activities that equip them with both generic and technical skills [10, 38, 47]. Moreover, the staff cultivates the culture of research and scholarship through dedicated activities throughout the programme years, such as writing essays, designing and presenting posters, an immersive viva exam in the final year, and regular journal clubs. In a broader context, staff need to align their teaching and leadership activities with the aims and objectives of the integrated, student-centred approach to shaping the entire curriculum and to creating a supportive learning environment for all students. These themes, derived from the firsthand insights of graduates, may inform programme stakeholders and offer specific practical recommendations for similar initiatives.

### Limitations

Although the findings may be transferable to similar transnational medical education contexts, the single-programme focus may limit applicability to other settings. Although the Qualtrics participation link was shared on the programme’s Facebook page, we cannot guarantee that all graduates viewed it, nor that the sample represented all cohorts. Self-selection and recall biases are recognised limitations. The predominance of domestic graduates may also have limited the diversity of international perspectives despite efforts to achieve variation in sampling. Future longitudinal follow-up studies could further explore graduates’ evolving career trajectories, while incorporating a survey component may help triangulate and integrate qualitative findings with broader quantitative perspectives.

To support future development of comparable TNE programmes, Table 2 presents key participant-informed recommendations derived from the thematic findings.

**Table 2:** Participant-Informed Recommendations for Transnational Medical Programmes

Key Area	Recommendations in the following areas:
Curriculum & training	• Expand procedural skills training opportunities.
	• Continue enhancing facilitation strategies in PBL.
	• Maintain ongoing foundational basic sciences during clinical placements.
	• Clarify student roles and expectations during clinical placements.
	• Address language barriers for non-Arabic speakers in clinical settings.
	• Continue strengthening communication skills training.
Assessment	• Support calibration of OSCE exams.
	• Provide mock assessments and access to an MCQ bank.
	.• Consider including external OSCE examiners.
Student Support & Learning Environment	• Continue supporting the cultural and academic growth of national and international students.
	• Continue promoting inclusive environments in teaching and administration.
	• Maintain and expand placement opportunities abroad
	• Streamline postgraduate application guidance and processes.
	• Support shared understanding of the licensing model and career pathways.

Note: Recommendations are raised by graduates from multiple cohorts; some have been addressed by the programme

### Participant-informed recommendations

These recommendations are informed by both participants’ experiences and the broader literature on transnational medical education, curriculum design, student support, and clinical training.

### Implications and future directions

The study offers actionable and realistic recommendations on curriculum, assessment, and learning environment. An integrated curriculum with early clinical exposure and international career preparation is essential to maintain. Inconsistencies in teaching and assessment practices should be addressed through regular faculty training programmes, especially around the dynamics of PBL and standardisation of MCQ and OSCE exams. The type of degree and its recognition should be clearly communicated to all stakeholders. Linguistic and cultural support for international students is crucial to the programme’s viability. Comparative research across programmes could validate findings and offer further insights. Future studies involving educators and employers could provide a more comprehensive perspective and evaluate the effectiveness and sustainability of improvements in PBL facilitation and assessment practices. Given graduates’ strong international career orientation and workforce mobility, future research should explore how transnational medical education programmes influence workforce distribution, retention, and migration patterns in both source and destination countries.

## Conclusion

This study provides new insights into how a transnational medical programme can transform its graduates, both personally and professionally. Drawing on their lived experiences, it highlights how an integrated, learner-centred curriculum fosters competent, resilient, and globally prepared graduates. Key contributing factors include small-group learning, early clinical exposure, international exchange opportunities, English-language instruction, cultural preparedness, and supportive academic staff. Based on these findings, the study offers practical recommendations for improving teaching, assessment and learning environment in similar programmes. Ultimately, it contributes to the design of more responsive transnational medical curricula that align with the diverse needs and expectations of their students and graduates.

## Supplementary Information

Supplementary Material 1.

Supplementary Material 2.

## Data Availability

All data generated or analysed during this study are included in this published article and its supplementary files (interview guide questions and COREQ checklist).

## Abbreviations

COREQ: Consolidated Criteria for Reporting Qualitative Research
HPE: Health Professions Education
IBC: International Branch Campus
IELTS: International English Language Testing System
MCQ: Multiple Choice Questions
MRCS: Membership of the Royal College of Surgeons
OET: Occupational English Test
OSCE: Objective Structured Clinical Examination
PBL: Problem-Based Learning
PLAB: Professional and Linguistic Assessments Board
TNE: Transnational Education
UKMLE: United Kingdom Medical Licensing Assessment
WFME: World Federation for Medical Education
WHO: World Health Organisation
