# Supplementary material for: Graduate perspectives on a transnational medical programme: a qualitative study of career impact
Source: BMC Med Educ. 2026 Jun 30;26:1479. doi: 10.1186/s12909-026-09693-8 (PMC13584353; doi:10.1186/s12909-026-09693-8)
Supplement: Supplementary file 2 [file 12909_2026_9693_MOESM1_ESM.docx]

Graduates’ Perceptions of a Transnational Undergraduate Medical Programme and Its Impact on Their Careers: A Qualitative Interview Study

Topic Guide Questions

1. Can I start by asking you about your career so far? Can you talk me through your career from medical school to now?

2. What made you choose the Mansoura Manchester Medical programme rather than

another conventional programme?

Prompts: family, friends, etc.

3. What did you think the differences were between the conventional and Manchester Mansoura Medical Programme when you were there? What do you think now when you look back?

Prompts: differences in goals, values, outcomes, and competencies.

4. How do you think this difference has influenced your practice?

5. How do you think this difference has influenced your career?

6. Please share any aspects of this programme that stand out for you, such as teaching

methods (case-based discussion), instructors, environment, feeling a connection to

another country, class sizes, anything else?

7. Final thoughts - Is there anything else that you would like to tell me about the

programme in terms of your experience/reflections?
